# Semantic querying of relational data for clinical intelligence: a semantic web services-based approach

**DOI:** 10.1186/2041-1480-4-9

**Published:** 2013-03-13

**Authors:** Alexandre Riazanov, Artjom Klein, Arash Shaban-Nejad, Gregory W Rose, Alan J Forster, David L Buckeridge, Christopher JO Baker

**Affiliations:** 1 Department of Computer Science and Applied Statistics, University of New Brunswick, Saint John, NB, Canada; 2 Department of Epidemiology, Biostatistics and Occupational Health, McGill University, Montreal, QC, Canada; 3 Department of Medicine, University of Ottawa, Ottawa, ON, Canada

## Abstract

**Background:**

Clinical Intelligence, as a research and engineering discipline, is dedicated to the development of tools for data analysis for the purposes of clinical research, surveillance, and effective health care management. *Self-service ad hoc querying* of clinical data is one desirable type of functionality. Since most of the data are currently stored in relational or similar form, ad hoc querying is problematic as it requires specialised technical skills and the knowledge of particular data schemas.

**Results:**

A possible solution is *semantic querying* where the user formulates queries in terms of domain ontologies that are much easier to navigate and comprehend than data schemas. In this article, we are exploring the possibility of using *SADI Semantic Web services* for semantic querying of clinical data. We have developed a prototype of a semantic querying infrastructure for the surveillance of, and research on, *hospital-acquired infections*.

**Conclusions:**

Our results suggest that SADI can support ad-hoc, self-service, semantic queries of relational data in a Clinical Intelligence context. The use of SADI compares favourably with approaches based on declarative semantic mappings from data schemas to ontologies, such as query rewriting and RDFizing by materialisation, because it can easily cope with situations when (i) some *computation* is required to turn relational data into RDF or OWL, e.g., to implement temporal reasoning, or (ii) integration with external data sources is necessary.

## Background

### Clinical intelligence and ad hoc querying of relational data

Clinical Intelligence (CI) is essentially Business Intelligence applied to clinical data, i.e., it is a business vertical and a research and engineering field aimed at the development of methods and tools for deriving insights from clinical data, required for research, surveillance and rational health care management (see, e.g., [1-5] to get a flavour of different directions of CI work). A typical example of CI is the use of patient records for selecting cohorts to be used in clinical trials of drugs and other treatments. Researchers could use CI tools on massive amounts of data to facilitate the discovery of knowledge that can improve existing treatment methods or help to define new treatments. Health care management can use CI to objectively evaluate the performance of hospital units or separate professionals and allocate resources more efficiently. The work of the Roundtable on Value and Science-Driven Health Care of the US Institute of Medicine, and in particular their work on the *Learning Healthcare System*[6] provides evidence of the need for such work.

One of the most useful modes of using clinical data is *ad hoc querying*: in many scenarios, such as clinical trial cohort selection, it’s very difficult to predict the kind of queries than need to be answered and preprogram them (see, e.g., eligibility criteria for various trials from [7]). Since the data is usually stored in relational or similar form, we can identify *ad hoc querying of relational data* as an important technical problem within CI.

### Semantic querying: problem and existing approaches

To be economical and therefore accessible, ad hoc querying has to be *self-service*, so that non-technical users – clinical researchers, surveillance practitioners, health care managers, etc. – can query clinical data directly, without help from database programmers. With the traditional database methods, this type of querying is problematic because writing correct queries requires good understanding of technical details of data schemas and the knowledge of query languages like SQL. Non-technical users almost never have such skills.

A viable solution to the problem, as demostrated, e.g., by the case study [4] conducted in Cleveland Clinic, is *semantic querying* (SQ) based on automatic application of domain knowledge written in the form of ontological axioms and, possibly, rules. Existing approaches to SQ (see, e.g., [8-11]) typically allow database programmers to define mappings between data schemas and domain knowledge bases, in the form of logical axioms or similar declarative constructs. The axioms map concrete data into virtual models usually based on RDF and OWL, so that the databases can be queried as RDF graphs or OWL ABoxes. End users formulate their queries *in the terminology of their domain*, without any knowledge of how the underlying data is structured. Semantic querying systems then use the semantic mappings to translate the semantic queries into queries directly executable on the data, or to translate the data itself to RDF.

Note that our assumption that semantic querying may be self-service is currently just a *vision* that may or may not be realised in the future, as commercial production-quality implementations are very scarce yet (a notable exception is the Cyc Analytic Environment by Cycorp Inc, used in [4]). As several other research projects, e.g., [4,8,12], our work aims to help to realise this vision.

The most basic form of semantic querying is querying semantic data, e.g., RDF, with languages like SPARQL, and our work is focused on this core problem. However, SPARQL querying is, by itself, not sufficient to fully realise the vision of self-service querying because it is difficult to expect that many non-technical users will be able to write SPARQL queries. More friendly graphical or keyword-based query interfaces, as in [4,12], have to be used on top of SPARQL. However, this problem is outside the scope of this article. Likewise, we do not discuss technical problems that have to be solved before semantic querying software can be deployed in clinical settings, such as security or profiled data access.

### When declarative mappings are not enough

Hypothetically, when the database design and the corresponding formalised domain terminologies reasonably fit together, the use of declarative semantic mappings may be both sufficient for many types of queries and cost effective, because declarative mappings are relatively easy to write, examine and edit. In most realistic clinical settings, however, declarative mappings cannot cope with all user requirements, specifically because *certain things cannot be done declaratively and require programming*.

One example of such a requirement, which is also highly relevant to our work, is the necessity of *temporal reasoning*. Many interesting queries to clinical data impose temporal constraints specifying how certain events, activities, or procedures of interest are temporally situated or temporally related to each other. For example, the user may be interested in retrieving only medical tests performed *prior* to some diagnoses of interest, within a *limited time period*.

Query engines processing such queries need to invoke some code doing essentially *temporal arithmetics*, i.e., comparison of concrete temporal values, or only slightly more complex temporal reasoning. This cannot be easily done by just extending the virtual RDF graphs representing the data if only declarative semantic mapping means are used – some computation has to happen dynamically during the query processing time.

We would like to emphasise that *temporal reasoning is only one, although very important, example* of a challenge for declarative mappings. In general, all data in the virtual RDF graph generated by a declarative mapping is constructed by simple transformations of data present in the database. Very often this data is not enough. A typical example is when some queries may require Body Mass Index (BMI) of patients, which are not necessarily directly stored in the database, but can be computed on the fly from patients’ weight and height, or various cardiovascular risk scores that can be computed on various patient attributes. Another typical problem related to Clinical Intelligence is the large amount of useful clinical information stored in free text form in various abstracts and reports. This kind of information can only be accessed via specialised text mining algorithms. However, in this article we focus our attention on the temporal reasoning problem as it seems to be required in a larger number of use cases and also demonstrated the capabilities of SADI well.

Another significant challenge for declarative approaches is integrating relational data with data from heterogeneous non-relational data sources using representations ranging from various ad hoc text file-based formats, popular with public biomedical database providers, to HTML content that has to be scraped. Querying such sources is inherently algorithmic and is beyond reach of current declarative mapping languages and implementations.

Note also that the axiomatic semantic mappings for real-life databases are often very complex, given that most non-trivial concepts expressed in the semantic schema need to be deduced or inferred from many data elements. This adds difficulty to their practical use.

### SADI and SHARE

The SADI framework [13] is a set of conventions for creating HTTP-based Semantic Web services that can be *automatically discovered and orchestrated*. SADI services consume RDF documents as input and produce RDF documents as output, which solves the syntactic interoperability problem as all services “speak” one language. This is also convenient for client programs that can leverage existing APIs for RDF to represent the data on which SADI services operate.

In brief, SADI services operate by attaching new properties to input URIs described in the input RDF document, and the most important feature of SADI is that these properties are fixed for each service. A declaration of these predicates, available online, constitutes a *semantic description* of the service. For example, if a service is declared with the predicate *is_performed_for* described in an ontology as a predicate linking diagnoses to patients, the client software knows that it can call the service to retrieve the patient for whom a given diagnosis was made.

The SADI framework uses the OWL class syntax to specify conditions on the input nodes a service consumes, and declare the predicates the service attaches. Such declarations of inputs and outputs of services enable completely automatic discovery and composition of SADI services (see, e.g., [13]). Practically, it means that one can have a query engine for SPARQL or a similar language that can answer queries by automatically discovering necessary SADI services and calling them. SHARE [14] is a proof-of-concept implementation of this approach. From the user point of view, it is a SPARQL engine that computes queries by picking and calling suitable SADI services from some registry. In a typical scenario, the user first looks up predicates he needs for his query, in the list of predicates declared as provided by SADI services in a registry, and also related classes and property predicates in the referenced ontologies. Then he uses the available concepts to form a regular SPARQL query, and sends it to a SHARE endpoint for execution.

For more technical details on SADI and SHARE, the reader is referred to [13], and Bioinformatics- and Chemoinformatics-related case studies can be found in [15-18].

### Article outline

These limitations of the declarative mapping-based approaches motivate us to look for other possibilities. In this article we explore an approach to semantic querying based on the use of Semantic Web services that can be automatically discovered, composed and invoked. More specifically, we are looking at the SADI [13] services as a possible medium for semantic querying of clinical data in relational form. We describe a prototype based on the SADI technology, we created to experiment with semantic querying, and report the results of a case study performed for several scenarios related to the surveillance of, and research on *hospital-acquired infections*, using an extract from a hospital datawarehouse.

## Methods

### The hydra query engine

We used SHARE in our early experiments on HAI-related semantic querying for Clinical Intelligence purposes, reported in [19]. At some point we reached the limits of SHARE performance – some of our queries were taking hours to complete, although the general amount of processed RDF data and the numbers of SADI service calls were moderate. Another feature of SHARE that hinders experimentation is the fact that the user has to wait until absolutely all answers to the query are computed before he can see any answers at all. Together with the low query execution speed, this makes query debugging very time consuming. For these reasons, we have opted to use both SHARE and Hydra [20] in our experiments.

Hydra mirrors SHARE, in terms of functionality, in that it executes queries over collections of SADI services. One notable extension is the ability of Hydra to draw data from multiple arbitrary SPARQL endpoints, giving end users the ability to use existing online data as input data. Unlike SHARE, which is Open Source, Hydra is a commercial prototype, based on a complex but scalable architecture and is being developed by IPSNP Computing Inc to address the lack of commercial quality clients for SADI. Despite being an early proof-of-architecture prototype, the system shows acceptable performance even on relatively complex queries in our use cases, and sets of services. A notable feature is that Hydra often returns first answers in quasi-real time, in minutes or even seconds, which facilitates experimentation with different forms of queries. In contrast SHARE returns answers in batch form when all possible services have responded. This can lead to lengthy delays or query timeouts. Two of the four experiments reported in this article were done exclusively with Hydra.

### Semantic querying of relational data with SADI

The SADI framework primarily facilitates the federated querying of multiple heterogeneous, distributed and autonomous data sources, such as online databases and algorithmic resources, as illustrated by several case studies [15-18]. The work presented here, however, explores a different avenue – using SADI services as a medium for semantic querying of single relational databases. If successful, our effort will add a new approach to the pool of existing practical methods for semantic querying of RDB, at least in the Clinical Intelligence context.

The general approach is to write a set of SADI services over a relational database that users would like to query semantically, and leverage the services collectively to answer ad hoc SPARQL queries, drawing the necessary data from the database. In this article we report on preliminary progress in implementing this idea on a clinical datawarehouse.

### Experiment settings: querying The Ottawa Hospital DW

We are testing our approach in a Clinical Intelligence scenario dedicated to the surveillance for, and research on Hospital-Acquired Infections (HAI). To this end, we are prototyping a SADI-based infrastructure for semantic querying of a relational database used by The Ottawa Hospital (TOH) and containing an extract from the large TOH datawarehouse accumulating data from the most important IT systems of the hospital (see, e.g., [21,22]). Our infrastructure consists of an *ontology* defining concepts suitable for reasoning about Hospital-Acquired Infections, and a number of *SADI services drawing data from the DB*, as well as several general purpose services dealing with information about drugs, diseases and infectious agents. In our experiments, we use SHARE and Hydra to run test queries over this network of SADI services. By using queries that HAI surveillance specialists or researchers may be interested in, we are trying to demonstrate the viability of our approach for real-life Clinical Intelligence tasks.

### Application: hospital-acquired infections

Infections acquired by patients in health care institutions are a serious practical problem as they result in thousands of deaths and hundreds of millions of dollars in additional expenses each year in Canada alone: at least one in twenty patients admitted to a Canadian hospital acquires an infection. Surveillance for, and research on HAI are essential to develop prevention methods, evaluate them and control their deployment. Currently, HAI surveillance based on clinical data is mostly manual and, as a consequence, is limited in scope and costly. Therefore, the use of adequate Clinical Intelligence tools promises to increase the effectiveness of HAI surveillance and research efforts and bring the cost down, which, in particular, justifies our effort to enable agile querying of HAI-related data with SADI.

#### Data

Our experimental efforts are focused on querying the TOH datawarehouse extract containing potentially HAI-related data from several clinical data sources, such as microbiology and clinical chemistry test results, information on drug prescriptions and surgical procedures, operating room information, and patient demographics and movement information. Our datawarehouse extract, referred to as DW throughout the rest of the article, contains information for 715 cardiac surgery patients, 6132 encounters, 12275 diagnoses and 6029 procedures, and has been previously used in a Clinical Intelligence effort [22] dedicated to cardiac surgical site infections. The data we used were selected from a cohort of all clean cardiac surgeries performed at the University of Ottawa Heart Institute in 2004-2007. In terms of design, the datawarehouse is a single Relational DB with tables representing patients, encounters, procedures and drug prescriptions, etc (the schema is available at [23]).

#### Target query types

From the perspective of HAI surveillance and research, we would like to be able to use the available data to answer HAI case identification and enumeration requests, questions aimed at identification or evaluation of HAI risk factors or causative factors, and questions aimed at identification or evaluation of diagnostic factors. Our target set of questions at this stage is as follows: 

• **Which patients diagnosed with surgical site infections (SSI) had older age as a risk factor?** Questions of this type allow surveillance practitioners to estimate the prevalence of particular risk factors.

• **How many patients were infected with methicillin-resistant Staphylococcus aureus (MRSA) in Quarter 1 of 2007?** Questions of this type allow health care managers to evaluate anti-HAI measures.

• **What patients received a diagnosis of sepsis within 30 days of being diagnosed with SSI?** Researchers may use such questions to develop methods for predicting complications of HAI.

• **How many SSI were diagnosed during quarter 1 of 2011 for patients who had undergone heart bypass surgery within 30 days prior to the SSI diagnoses?** Questions of this type allow to estimate the incidence of HAI for particular categories of patients.

• **How many Catheter Associated Urinary Tract Infection (CAUTI) incidents in January 2011 were diagnosed within 30 days following procedures involving Foley catheters?** Surveillance personnel can ask such questions to evaluate the risks associated with new types of equipment.

• **Which patients were diagnosed with SSI while they were taking corticosteroids systemically?** Questions of this type make it possible to identify patient risk groups based on the types of medications they take.

• **How many cases of hypoxia (decreased oxygen supply) were not accompanied by hypoalbuminemia (reduced serum albumin concentration) and SSI diagnoses?** Researchers can ask questions of this type allow to discover interactions between various risk factors.

• **How many CAUTI diagnoses were made within 7 days from the time a test indicated the patients’ serum glucose level was between 7.0 and 7.5 mmol/L?** Questions of this type allow to identify ’dose-response’ relationships between numerical findings of medical tests and the risk of HAI.

• **How many diabetic patients were diagnosed with SSI?** In general, such questions are aimed at identifying comorbidity factors for HAI.

• **Elevation of which proteins was indicated by blood test results within 7 days prior to the patients being diagnosed with HAI?** Questions of this type help to discover new diagnostic factors for HAI.

• **Which patients using drugs with anti-inflammatory side effects, had an operation and were diagnosed post-operatic abscess formation not detected preciously by a tomography?** Questions of this type allow to identify weaknesses of existing diagnostic methods.

These sample questions illustrate the *ad hoc* nature of the queries we would like to compute over our datawarehouse and, in general, over similar sources of clinical data. Although such questions can be implemented with SQL queries, this approach is uneconomical as it requires involvement of database programmers. Ideally, we would like to enable non-technical users, such as surveillance practitioners and researchers, to write ad hoc queries by themselves. The solution we are advocating is *semantic querying* where users formulate queries in terms of domain ontologies that are much easier to navigate and comprehend than relational database schemas.

Other kinds of requirements associated with our use cases are either trivial, e.g., it’s clear that extreme querying speed is not necessary, or can only be identified through experimental deployment of some software prototypes, for which our research is still in too early a stage.

### Ethical consent

The work reported in this paper was carried out, in part, using data from a secondary source previously made available, citation [22], and no additional patient consent or ethical approval was necessary.

The HAIKU project was reviewed and approved by the Institutional Review Boards at the OHRI and at McGill University. Since we are using individual patient records (even if personally identifying information is removed), IRB approval was required and was obtained.

## Results

### Outline of the infrastructure

#### HAI Ontology

In general, semantic querying implies the use of formalised terminologies as sources of query primitives. For this reason, we are developing the HAI Ontology (HAIO) [24,25] that defines a number of HAI-specific concepts, such as *Surgical_site_infection* and *Hospital-acquired_tuberculosis*, adds a small hierarchy of general health care-related concepts, such as *Disease* and *Medical_test*, and aligns the resulting ontology with a number of third party ontologies, both general and specialised.

The ontology design is fairly straightforward. Its core is a hierarchy of classes formalising concepts from the HAI domain and a flat hierarchy of object properties representing relations of interest between entities, such as *goes_through* to link patients to the operative procedures they undergo, and *is_performed_for* to link diagnoses to the patients they were made for.

We are using the Semanticscience Integrated Ontology (SIO) [26] as the upper ontology to provide access to our classes and properties via more general classes and properties. Currently, HAIO is undergoing the alignment to SIO and eventually most of our primitives will be mapped to appropriate places in the SIO hierarchy. We are also removing datatype properties to follow the SIO convention that the only datatype property is ’has value’ (SIO_000300) and all data-valued attributes are represented as individuals.

Apart from SIO, we use several other separate ontologies most of which are large biomedical nomenclatures. For example, to be able to use the Canadian version of the ICD-10 [27] nomenclature of diseases, we have created an OWL version [28] of the ICD-10 hierarchy. Also, to be able to reason about drugs, we created OWL versions [28] of the Anatomical Therapeutic Chemical (ATC) classification that provides a hierarchy of active ingredients, and of the Canadian Drug Identification Number (DIN) nomenclature. We also developed the Extra Simple Time Ontology (ESTO) [29], to be able to specify temporal coordinates of activities and events and compare them, which will be discussed in more detail in Section *Main difficulty: temporal reasoning*.

**Figure 1:**
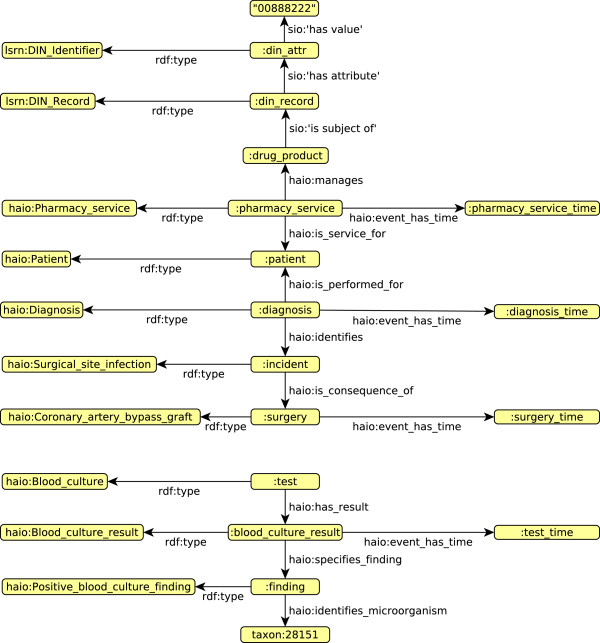
**Figure**1**HAI Ontology modelling fragment.** This RDF graph illustrates how HAI Ontology concepts can be used to semantically model data from the datawarehouse in particular, and from the HAI domain in general.

To give the reader some flavour of the kind of modelling that HAIO supports, we provide an RDF example in Figure 1. This RDF graph is a description of the patient identified as *:patient*. The node *:diagnosis* and the subgraph around it represents a diagnosis linked to *:patient* with the predicate *haio:is_performed_for*. The diagnosed disease is identified as *:incident*. Since the disease is a surgical site infection, it must be a consequence of an operative procedure. In our example, the procedure *:surgery* is an instance of *haio:Coronary_artery_bypass_graft*. The rest of the example describes a blood culture test *:test* that revealed the presence of *Serratia proteamaculans* in the patient’s blood, and a prescription of a drug (DIN 00888222) to the patient.

#### Mapping the DW schema to HAIO

A key ingredient of any implementation of semantic querying over a relational database is the semantic mapping of the database schema to the corresponding ontologies, specifying, in one form or another, how the database contents are modelled semantically, e.g., as a virtual RDF graph. We are mapping the relevant parts of the Ottawa Hospital DW schema [23] to the HAI Ontology. This is done by specifying how classes and properties from HAIO are populated, based on the contents of relevant tables in the DW. In all cases we have encountered so far, the population of the RDF graph representing an ABox for HAIO, can be done by running one or more SQL queries over the DW and converting the results of the SQL queries into RDF by creating necessary resource URIs and data literals, and asserting RDF triples on them.We illustrate this by the following examples.

##### Example 1

**populating*haio:Patient*.** Populating the class *haio:Patient* with instances amounts to identifying all patients in our DW and assigning URIs to them. Note that the patient URI generation from the DW keys for patients must be *invertible*, so that SADI services that accept patient URIs as input are able to identify the relevant DW records. This consideration applies to all URI generation schemes in our experiments.

The datawarehouse schema has table *Npatient* containing basic information about all patients, such as names, dates of birth, medical record numbers, etc. The table’s primary key consists of one integer-valued attribute *patWID* which is used throughout the datawarehouse to identify patients. We simply map these integer patient IDs to URIs as follows. Suppose, the *patWID* value is 123. The corresponding URI is haiku:Patient_by_patWID?wid=123, where the namespace haiku corresponds to http://cbakerlab.unbsj.ca:8080/haiku/.

In general, we simply attach the *patWID* as a query parameter in the URI, so that the URI can be viewed as a function of the *patWID*. The function is easily invertible since the *patWID* value can be extracted as the value of the *wid* parameter of a given patient URI. We use this primary key-to-URI mapping scheme for most entities that have to be identified with URIs in the virtual HAIO ABox, such as diagnoses, diseases, medical tests, drug prescriptions, etc.

##### Example 2

**populating*haio:is_performed_for* and*haio:identifies*.** Our goal now is to specify how patients are linked to their diagnoses and disease incidents, based on the DW contents. The DW has a table *NhrDiagnosis* with information about diagnoses, but its records do not directly specify the patients. However, *NhrDiagnosis* uses foreign key *hdgHraEncWID* to table *Nencounter* representing encounters, where the attribute *encPatWID* is the foreign key to *Npatient*. The attribute *NhrDiagnosis.hdgCd* also specifies the ICD-10 [27] code of the disease. The following SQL query can be used to enumerate triples containing patient and diagnosis IDs and disease codes:

The corresponding part of the virtual RDF graph can be formed by asserting

for each triple (*patientID,diagnosisID,diseaseCode*). Note that in this pseudo-RDF *Diagnosis_by_hdgWID*, *Patient_by_patWID*, and *Disease_by_diagnosis* are functions constructing URIs from numeric and string-valued parameters similarly to how it was done for patient URIs in the previous example. The function *disease_class_by_ICD10* represents a slightly different case as it constructs disease class URIs according to our OWL version of the ICD-10 nomenclature (Canadian edition). For example, *diseaseCode=*A40 (“Streptococcal septicaemia”) is mapped to the URI ont:icd10ca.owl#A40, where the namespace ont is defined as http://cbakerlab.unbsj.ca:8080/ontologies/.

It is important to clarify that, although we have presented an SQL query, we do not actually execute it and construct explicit RDF from the results. The SQL and the pseudo-RDF are only written *to document our understanding* of how the data is mapped. Our SADI services implementing the mapping use slightly different SQL queries because a service always has some piece of data-e.g., a patient URI or diagnosis URI – given to them as input.

In fact, we don’t even literally write SQL to document our semantic mapping. We use PSOA RuleML [30] – an expressive rule language – to express the semantic mapping. For example, the population of *haio:is_performed_for* and *haio:identifies* is captured by the following PSOA RuleML rule:

Lines 9–13 essentially represent the SQL query given above, and lines 1-6 and 14–17 capture the meaning of the pseudo-RDF. We provide the rule only as an illustration, so readers interested in details of PSOA RuleML are referred to [30].

We would like to avoid making a false impression that semantic mapping of relational data schemas is simple. Typical challenges are, e.g., associated with insufficiently expressive ontologies and the general difficulty of semantic modelling of data. Since this activity deserves a separate investigation, in this article we restrict ourselves with a relatively brief discussion of it.

#### SADI services

The core part of our semantic querying infrastructure will be a set of SADI services providing several HAIO predicates according to the semantic mapping discussed above, by drawing the necessary data from the DW. The general scheme for the execution of these services is as follows. The code of a service (i) accepts some input RDF describing certain entity identified with a URI, (ii) decomposes the description into a set of values and embeds them as parameters in one or more predefined service-specific SQL queries, (iii) runs the SQL queries over the DW, (iv) converts the result values to a new RDF description of the input entity and returns the description as its output.

To illustrate this scheme, we anatomise one of the already written services – *getDiagnosisByPatient* – that enumerates all known diagnoses for a specified patient. It implements a part of the semantic mapping defined in Example 2 from the previous section. The input class of the service is just *haio:Patient* and any URI qualified as an instance of *haio:Patient* is legitimate input to the service. The service only processes URIs of the form described in Example 1, in a meaningful way. As soon as it receives such a URI, it extracts the integer DW key for the patient from the URI. The service then executes the SQL query

where the parameter “?” is replaced with the patient key value. Then, for each value of *diagnosisID* in the result relation, the service constructs the corresponding diagnosis URI (as *Diagnosis_by_hdgWID(diagnosisID)*), qualifies it as an instance of *haio:Diagnosis* and attaches it as the value of the property *haio:patient_has_diagnosis* to the patient URI. The output class of the service, in Protégé syntax, is *(patient_has_diagnosis***some***Diagnosis)*. Note that, in HAIO, the property *haio:patient_has_diagnosis* is defined as the inverse for *haio:is_performed_for*, so the service indeed implements a part of the mapping from Example 2.

To illustrate the composition of SADI services, consider another service *getDiagnosisInfo* which annotates a specified diagnosis URI with the disease incident diagnosed, through the predicate *haio:identifies*, and with the time when the diagnosis was made, through *haio:situation_has_time*. So the input class of *getDiagnosisInfo* is *haio:Diagnosis* and the output class is *(identifies***some***Disease)***and***(situation_has_time***some***FullyDefinedTimeInterval)*. Because a part of the output from *getDiagnosisByPatient* is a URI explicitly qualified as an instance of *haio:Diagnosis*, this URI can be directly submitted as input to *getDiagnosisInfo*. Practically, if some client program has an instance *:patient* of *haio:Patient* and “wants” to know disease incidents diagnosed for the patient, it can first call *getDiagnosisByPatient* to retrieve the relevant diagnoses, and then call *getDiagnosisInfo* on these diagnosis URIs.

Overall, we currently have fourteen finished services similar to *getDiagnosisByPatient* and *getDiagnosisInfo*, and this number is likely to double as our infrastructure matures. In terms of the scope, the services compute, or will compute, instances of HAIO classes for patients, diagnoses, procedures, drug prescriptions, medical tests, etc, and provide information about such entities, such as patient names, time of procedures, links between patients and diagnoses and medical tests, etc. In terms of the complexity, most of the services we already have are similar or slightly more complex than *getDiagnosisByPatient*: typically, the service execution amounts to instantiation of a few SQL query templates and a fairly straightforward transformation of the query results into output RDF. This type of programming can be easily done by moderately skilled DB administrators or programmers. Finally, in addition to SADI services working on the DW and created specifically for HAIKU – our project on ontology-supported HAI surveillance, we are using a number of external general purpose SADI services, dealing mostly with drug and disease information, and a set of services for temporal reasoning, that will be discussed in the *Temporal Reasoning* section.

#### Querying with Hydra and SHARE

The end users of our infrastructure will access the SADI services and, through the services, the underlying data in the DW, by querying our network of SADI services in the SPARQL language. For our current experiments, we have been using Hydra and, to some extent, SHARE – two prototypes that implement such functionality.

To enable service discovery, we register all relevant services in a private registry, and the query engines are configured to query this registry for services providing particular predicates necessary to resolve SPARQL queries being executed. Users interact with the DW only via the semantic front end. They need not know anything about the DW schema or write SQL queries, and have no direct access to the relational data. To form queries, users look up concepts of interest in HAIO and accompanying ontologies, including the ontologies referred to from the descriptions of non HAIKU-specific services in the registry. For browsing convenience, we have created an umbrella ontology [31] that simply imports all relevant ontologies, can be browsed with the help of standard ontology editors such as Protégé, and works, effectively, as a kind of “semantic schema” for the DW. The user finds relevant classes and properties in the ontology, and uses their URIs to manually form a SPARQL query. Note that this is only a temporary solution to facilitate early stage experimentation – in the future we will look for more productive ways to create queries.

Finally, we would like to mention that, in contrast with SPARQL querying of static RDF triplestores, Hydra, as well as SHARE, can only start calling services if it already has some “seed” data. This seed data comes from RDF files specified with their URLs in FROM sections of queries. Examples of this will be given in the evaluation section. Hydra can also get the seed data from one or more specified SPARQL endpoints, but we did not need this feature in our experiments.

### Major difficulty: temporal reasoning

Our very first efforts to analyse the problem of semantic querying for the purposes of HAI surveillance and research revealed that most questions we would like to answer based on the DW contents, have temporal components. A simple example is question (2) from the section introducing our HAI application: *“How many incidents of diseases caused by meticillin-resistant Staphylococcus aureus bacteria, were diagnosed in Quarter 1 of 2007?”*. The temporal constraint embedded in this question requires the disease incidents to be diagnosed between Jan 1 and Mar 31, 2007. A slightly more complex example is question (6) *“Which patients were diagnosed with SSI while they were taking corticosteroids systemically?”*. In correct answers, the times of surgical site infection diagnoses must fall within some administered periods for corticosteroids for the same patients. To accommodate this kind of requirements, we have to solve two problems.

First, we need a way to define temporal entities, such as time intervals and instants, and to express relations between them, i.e., we need a time ontology. We also need predicates in HAIO to link activity- and event-like entities, such as diagnoses and procedures, to their temporal coordinates. These features should enable our SADI service to return temporal information for various entities, as well as enable users to capture temporal constraints in SPARQL.

Second, we need to be able to resolve temporal constraints in queries, which requires *temporal reasoning* at least in the form of temporal arithmetics on the temporal entities that can be defined.

#### Time ontology

As a solution to the first problem, we have created the Extra Simple Time Ontology (ESTO) [29]. We can define instants by specifying their XSD *dateTime* value:

We can define proper time intervals by specifying their ends:

We also have two dedicated URIs denoting infinite points in the past and future. Time intervals in our model are defined by specifying their ends as instants. Infinite time intervals can be specified by using infinities as their ends. Duration values can be associated with intervals and instants are treated as intervals of zero duration.

The core of ESTO is a set of properties for comparison of time intervals. The main properties are just the Allen’s temporal predicates [32]. For example, we can check in a query if a time interval is subsumed by another interval by using Allen’s predicate *during*:

#### Temporal reasoning

Several of our SADI services supply temporal information on various entities. For example, service *getDiagnosisInfo* provides, among others, predicate *haio:situation_has_time* specifying the instant when the corresponding diagnosis was made. Likewise, service *getPharmacyServiceInfo* attaches a time interval as an instance of *haio:Administered_period* via the predicate *haio:has_specification*. We can have a query with the following fragment:

Assuming that Hydra or SHARE instantiates the variables *?DiagnosisTime* and *?AdministeredPeriod* with some time interval URIs, e.g., by calling *getDiagnosisInfo* and *getPharmacyserviceInfo*, it only needs to *test* the predicate *esto:during* on these values. However, there is no SADI specification-compliant way to do this because SADI framework currently only supports services that attach predicates and supports neither services that would *test* specific *predicates* on specified arguments, nor services that would accept *more than one input parameter* and establish some facts about them. These SADI deficiencies motivated us to look for a workaround.

One obvious possibility is not to use high-level predicates like *haio:during*. We could replace our query fragment with another fragment that invokes the built-in arithmetics on XSD dateTime values via the SPARQL FILTER construct:

The main problem with this solution is that it requires significant extra work and too much technical knowledge from the end user, which is exactly what we are trying to avoid by using semantic querying. Recall that our ultimate goal is to facilitate self-service querying. We are assuming that using high-level temporal predicates like *esto:during* with obvious semantics would not present any extra difficulty for non-technical users, whereas the FILTER-based solution would require them to remember the details of the time period representation and make the query composition more error prone. So, we chose to try different solutions that avoid complicating queries by leveraging one of the main features of SADI – the ability of SADI services to do *computation* behind semantic interfaces.

Before we describe the solutions, we would like to briefly discuss possibilities of implementing temporal reasoning more complex than temporal arithmetics. In one of the first versions of our infrastructure we experimented with SADI services that did not require the input intervals to be *fully defined*, i.e., specified with their ends given as specific date-time values. These services could also work on intervals that are only partially defined by by specifying how they relate to other intervals, by implementing, in a limited manner, the axiomatic semantics of Allen’s temporal predicates [32]. We skip the discussion of this possibility primarily because temporal arithmetics is sufficient for our goal of illustrating SADI capabilities, but also because none of our use cases so far required such reasoning.

#### Query ID-based solution

Our first attempt to embed temporal reasoning into the DW querying resulted in the creation of a *temporal reasoner* in the form of a *set of SADI services*, providing Allen’s predicates from ESTO and working on a *shared cache of time intervals*. We will explain this by anatomising two of the services – *getTimeIntervalsDuringTimeInterval* and *getTimeIntervalsContainingTimeInterval* – providing, respectively, Allen’s predicates *contains* and *during*, that are inverse to each other. Suppose SHARE or Hydra instantiates the variables *?DiagnosisTime* and *?AdministeredPeriod* with *:diag_time* and *:adm_period* respectively, and then decides to call *getTimeIntervalsContainingTimeInterval* on the description of *:diag_time* to identify all intervals, known to the temporal reasoner, that can be attached to *:diag_time* via *esto:during*. It’s possible that *:adm_period* is *not yet known* to the temporal reasoner, i.e., it is not yet in the cache, and the call to *getTimeIntervalsContainingTimeInterval* will not confirm

which is required to resolve the temporal constraint

in the query. However, the temporal reasoner will now remember the interval *:diag_time* and, when the other service *getTimeIntervalsDuringTimeInterval* is called on *:adm_period*, it will attach *esto:contains :diag_time* to *:adm_period*, which is sufficient to resolve the constraint because *esto:contains* is inverse to *esto:during*.

Obviously, this approach cannot be practical unless there is a way to segment the time interval cache in the temporal reasoner into sub-caches corresponding to different queries because otherwise intervals from different queries will be returned by temporal services and pollute the working memory of the query engine. SADI does not support any notion of query IDs, so we had to devise a workaround for this.

To this end, we created a small ontology [33] providing class *cont:Context* and property *cont:hasContext*, where *cont* is an abbreviation for the ontology namespace. Instances of *cont:Context* can represent query IDs and *cont:hasContext* links various resources to the IDs of the relevant queries. We assume that all SADI services to be used for querying over the HAI DW can accept inputs with attached *cont:hasContext* and *propagate* the query IDs to all URIs in the output. We also require users to attach a unique query ID to all URIs mentioned in a query and in seed data. These conventions guarantee that all time intervals submitted to the temporal reasoner services carry query IDs with them and the services use this IDs to segment the cache.

#### Candidate list-based solution

Although the query ID-based approach solves the problem in principle, it suffers from two major drawbacks. First, the requirement to propagate query IDs imposed on services strongly hinders the use of external SADI services in queries: the query engine can only use an external service without query ID propagation if the data returned by the service does not have to be submitted as input to services requiring *cont:hasContext*. Second, the temporal reasoning services based on comparing input time intervals with absolutely all known time intervals, including instances, tend to produce too large outputs. In one of our experiments involving processing several thousands of diagnoses from the DW, a batch call to *getTimeIntervalsDuringTimeInterval* produced 750,000 RDF triples which took several minutes to transmit from the service to the query engine over the Internet. These problems motivated us to look for a more efficient solution.

Hydra experimentally supports the following *extension* to the SADI framework. When it realises that testing some predicate, say *esto:contains*, on two specific resources, say *t*_1_ and *t*_2_, could help to satisfy the query being executed, it asserts an additional fact *t*_1_*ext*:*isComparedToObjectt*_2_ in its working memory, where *ext:isComparedToObject* is a special predicate introduced specifically for this purpose. This predicate can be used to implement *predicate-testing services* as follows. Suppose, we want a SADI service testing the predicate *esto:contains*, i.e., somehow given two time interval descriptions *x* and *y*, the service has to compare them and if the condition *xesto*:*containsy* holds, report this. With the help of *ext:isComparedToObject*, the pair of values can be submitted by making *x* the main input node and adding *xext*:*isComparedToObjecty* to its description. Our service, say *getTimeIntervalsDuringTimeInterval*, having received *x*, looks for all resources *y*_*i*_ attached to it via *ext:isComparedToObject* and compares *x* to them by doing temporal arithmetics, e.g., by extracting specific values for interval ends and comparing them with appropriate Java methods. For all *y*_*j*_ satisfying *xesto*:*containsy*_*j*_, the service adds the triple *xesto*:*containsy*_*j*_ to the output description of *x*. In other words, the client program sends a list of *candidatesy*_*i*_ to the service by attaching them via *ext:isComparedToObject*, and the service *selects* the output property values from the provide list rather than computes completely new values.

This *output candidate list*-based solution is free from the drawbacks of the query ID-based solution discussed above. First, only the services that are meant to be predicate-testing services need to support the predicate *ext:isComparedToObject* in the input. No other services are affected and any external services can be used as long as they implement compatible data modelling. Second, there is no combinatorial explosion in the outputs caused by irrelevant comparisons of many temporal entities to many temporal entities: services supporting *ext:isComparedToObject* only perform comparisons that are relevant to the query and directly requested by the query engine. The cost of this elegance and corresponding performance gain is on-compliance with the current SADI specification: services based on *ext:isComparedToObject* will be useless with clients not supporting this special predicate. However, once this solution is tested in more scenarios, the corresponding extension to the SADI framework will be officially proposed.

## Evaluation: pilot use cases

To empirically test the utility of our SADI-based approach and infrastructure for semantic querying of The Ottawa Hospital DW, we formulated four SPARQL queries implementing different questions from the list in the *Target query types* section above. Two of the queries were executed with SHARE, as was reported earlier in [19], albeit using service implementations modified to only return a small part of the generated output data in order to increase SHARE performance. The other two queries were executed with Hydra with full versions of the services. Since our research task is to demonstrate the *principle possibility* of using SADI Semantic Web services for semantic querying of clinical data, *we do not pay much attention to the query performance* yet. We would only like to briefly mention that the two queries executed with SHARE took several hours and Hydra took between 20 minutes and 1 hour (on a commodity laptop) to compute all answers of the two queries it was tested on. Although the first answers are obtained by Hydra in minutes, this kind of performance is still unlikely to be sufficient in practice. However, note that Hydra is in its infancy and its performance is expected to improve by several orders of magnitude when it matures into a commercial quality system.

Let us now discuss the implemented queries.

### Use case 1

Our first query intended to implement Question (9) *How many diabetic patients were diagnosed with surgical site infections (SSI)?*, is as follows:

Consider the FROM clauses first. File *query1_as_context.rdf* contains a description of a query ID URI as an instance of *cont:Context* and *operative_procedure.rdf* attaches the query ID to the URI *haio:Operative_procedure* to make it comply with the conventions described in the *Temporal Reasoning* section. This is not necessary when the query is executed with Hydra and predicate-testing versions of the temporal reasoning services.

Let us now discuss the ontological primitives that form the query. In line 5, we use *haio:Operative_procedure* to specify the class of all surgeries, and *haio:procedure_type_has_instance* to link it to its instances. We use *haio:has_consequence* to link surgeries to their complications and *haio:disease_has_type*, a specialisation of *rdf:type*, to test if a complication is an SSI. Predicate *haio:is_done_on* is sufficient to retrieve the patients who undergo the corresponding surgeries. Predicates *haio:patient_has_diagnosis* and *haio:identifies* serve to retrieve diagnoses and diseases being diagnosed, and *haio:disease_has_type* checks if the disease is of the type *haio:Diabetes_mellitus*. In lines 16–18, we retrieve the times of the surgery and diabetes diagnosis, and compare them. In lines 21–24, we identify the interval between those times and test its duration.

The query execution starts with a call to the service *getProcedureByClass* that enumerates URIs of all procedures in the DW whose CCI [34] codes indicate that they are of the specified procedure class. Line 7 in the query is resolved with a call to *getSSIBySurgery* that retrieves incidents of infections that are suspected complication of the specified operative procedure. For this purpose, the current service prototype extracts from the DW all diagnoses made for the same patient within 30 days from the operation, and selects only incidents with ICD-10 codes indicating complications of surgeries. Service *getPatientByProcedure* instantiates *?Patient* in line 9, then *getDiagnosisByPatient* instantiates *?DiabetesIncident* with all diseases the patient was diagnosed with. These services use fairly obvious SQL queries to the DW. Testing that *?DiabetesIncident* is a diabetes mellitus (lines 12–13) is done by calling *getDiseaseClass* that will assign subclasses of *haio:Disease* based on the ICD-10 codes from the DW table for diagnoses, although the current prototype can only deal with *haio:Diabetes_mellitus*. Alternatively, to improve performance, we could implement it as a predicate-testing service supporting the predicate *ext:isComparedToObject*. Lines 16-17 are resolved with *getDiagnosisInfo* and *getProcedureInfo* that retrieve the necessary times from the records of encounters referred to from the DW tables for diagnoses and procedures. Lines 18–23 are resolved with several calls to temporal reasoning services.

Finally, to illustrate the benefits of semantic querying, we provide a simplified SQL query equivalent to our SPARQL query:

It is clear that the user composing such an SQL query would have to know some SQL and, more importantly, be able to navigate the complex DW schema and learn to use rather non-mnemonic table and attribute identifiers. The query composition process is also quite error prone, e.g., the user would have to be very accurate not to omit some of the diabetes or SSI-related code prefixes. In contrtast, the SPARQL query hides all such technicalities behind a relatively simple and mnemonic notation. In semantic querying, the burden of sorting out the technicalities lies on the engineers implementing it and, although it may require a considerable effort, the results can be later reused by multiple users in multiple scenarios.

### Use case 2

Our second query, implementing Question (6) *What patients were diagnosed with SSI while they were taking corticosteroids systemically?*, is as follows:

The file *corticosteroids.rdf* simply attaches a query ID to the URI for the class ’corticosteroids for systemic use’ from the ATC ontology. The line with *hasSubClass* is resolved with a call to http://cbakerlab.unbsj.ca:8080/atc-sadi/getSubclassByATCDrugClass, which is an external, i.e., non DW-specific service. Note, however, that we had to modify the service code for our experiments with SHARE, to make it propagate query IDs if they are provided in the input. This is not necessary for temporal reasoning with Hydra, as explained in the section on temporal reasoning.

In lines 9–15, we use SIO primitives and LSRN [35] identifiers to leverage the SIO modeling for database records. In our situation, an ATC drug class is “referred to by” a DIN record which has a DIN ID as an attribute and whose subject is a drug product, identified with a URI. Lines 9–13 are resolved with another general purpose service http://cbakerlab.unbsj.ca:8080/din-sadi/getDINByATCDrugClass, also conforming to the context propagation convention.

In the first half of line 16, we link pharmacy services to the drug product via *haio:manages*. It is resolved, together with line 15, by a call to service *getPharmacyServiceByDIN*, which obtains the necessary data from table *NphmIngredient* in the DW containing DINs of prescribed drugs as a separate attribute.

The predicate *haio:is_service_for* links services to the corresponding patients and is provided by *getPatientByPharmacyService*, trivially implemented as a join of appropriate DW tables. Primitives from the remaining lines, and the services implementing them, have already been discussed.

### Use case 3

The following query implements Question (2) *How many patients were infected with methicillin-resistant Staphylococcus aureus (MRSA) in Quarter 1 of 2007?*:

The files in the FROM clauses contain RDF descriptions of the time interval *esto:quarter1_2007* representing Quarter 1 of 2007, and a resource representing a name with the value “Staphylococcus aureus N315 (MRSA/VSSA)”.

The main feature distinguishing this query from the two previous queries is the extensive use of external SADI services: all conditions in Lines 12–35 require calls to such services. The external (not based on our DW) services used by Hydra provide the following functionality: (a) finding a KEGG Organism record and ID by the bacteria name (Lines 12–20), (b) retrieving KEGG DISEASE records and IDs for conditions caused by organisms specified with their KEGG Organism IDs (Lines 22–29), and (c) ICD-10 disease classes corresponding to diseases specified with their KEGG DISEASE IDs (Lines 31–35).

Note that by using the external services we go far beyond what is possible with SQL querying of the DW. This use case demonstrated that the data integration aspect of SADI is an important advantage. However, we refrain from a more thorough discussion of this because data integration with SADI has been studied in several specialised projects (see, e.g., [15-18]).

### Use case 4

The following query implements Question (3) *What patients received a diagnosis of sepsis within 30 days of being diagnosed with SSI?*:

In the previous query in Use case 3, a specific bacteria name “Staphylococcus aureus N315 (MRSA/VSSA)” was used, which assumed that the user somehow knows the precise name from the KEGG Organism nomenclature. Sometimes this assumption is too strong and we want the user to be able to formulate less precise questions as SPARQL queries. The current use case illustrates this: we assume that the user is interested in various disease names mentioning “sepsis”. The work of finding such names is done by an external SADI service that resolves the conditions in lines 8–16. Other external SADI services used by Hydra provide the following functionality: (a) finding URIs of disease classes from our ICD-10 ontology (see [28]), given precise names of the disease classes, (b) enumerating subclasses of a disease class specified with an ICD-10 URI – this is necessary because potentially a disease class related to sepsis may have subclasses not having “sepsis” in their names, and (c) mapping ICD-10 disease class URIs to ICD-10 records and IDs.

## Related work

We are not aware of any work on semantic querying of relational databases via Semantic Web services. However, there have been a lot of attempts, since as early as 1993 [36], to implement semantic querying of RDB. We cannot give a comprehensive overview of all related publications, so we will focus our discussion on the key concepts and methods.

### Declarative semantic mappings

Practically all existing approaches to semantic querying of RDB use some sort of declarative mappings from source relational data schemas to target semantic schemas. The most popular state-of-the-art approaches use OWL ontologies or RDF vocabularies as target schemas (see, e.g., [8-10,36]), but different approaches have also been tried – e.g., in [37] the target semantic schema is essentially a regular relational schema with tables corresponding to well-defined concepts with mnemonic names, that are easier to understand for a non-programmer user than the source schema.

The purpose of the semantic mappings is to define an interpretation of data from an RDB in terms of the concepts and relations defined in the target schema. The implementations then use these mappings to either completely translate the relational data or by applying them in query time, which will be discussed below in more detail. Note that we are also using declarative mappings to *document* the semantic modelling of our data (see Section *Mapping the DW schema to HAIO* above).

Declarative mappings are expressed in the form of axioms postulating how facts (rows) in the source RDB are related to instances and facts in the target schema. Typically, the axioms are Horn rules in some specific syntactic form, such as OWL axioms, as in [9], or SPARQL CONSTRUCT queries, as in SWObjects [38]. However, there are approaches that support much more expressive semantic mapping and query languages: e.g., [11,39] can deal with any first-order logic axioms.

Declarative semantic mappings for RDB are often created manually by programmers. D2R [40] and Virtuoso [41] can also automatically generate a preliminary mapping that can be later modified by the user, e.g., in order to align it with the target schema. The RDB2RDF Working Group [42] at W3C is taking this approach further by standartising both the language R2RML for mapping RDB to RDF, and defining a standard way to map schemas (*Direct Mapping*). The virtual direct graph produced by applying the Direct Mapping to a relational schema can be further mapped to the target schema by additional axioms, e.g., SWObjects implements SPARQL CONSTRUCT for this purpose.

The declarative nature of axiom-like mappings makes them relatively easy to create and maintain, at least compared to our approach based on hard-coding mappings in Java or other programming languages. However, declarative mappings have some limitations that are hard to overcome practically: certain things are much easier to define programmatically than axiomatically, due to natural limitations of mapping languages and implementations. For example, defining Allen’s predicates for temporal comparison in a practical fashion, or defining the Body Mass Index function is likely to be challenging with some of the existing declarative approaches. Mapping and retrieving data from non-relational sources is even harder. Overall, the SADI-based approach is more flexible – even very tricky relationships between the source and target schemas can be captured by programming.

### Materialisation

Defining a semantic mapping for an RDB creates a *virtual database* instantiating the target semantic schema. Since most of the state-of-the-art approaches map relational data to RDF, for simplicity we can speak about *virtual RDF graphs*. A straight forward way to use a virtual RDF graph, e.g., for query answering, is to actually *materialise* it, i.e., to create a triplestore representing it and make it available for querying or browsing. This approach is implemented at least in Triplify [43] and Minerva [44].

An obvious advantage of this approach is that even large RDF graphs materialised in a triplestore can be processed very efficiently, due to the availability of mature highly optimised implementations. One obvious limitation of this approach is its inability to deal with live data: the data has to be transformed into RDF before it can be queried. However, most Clinical Intelligence scenarios other than surveillance, don’t require querying live data.

A more significant practical problem is the limited scope of the data that can be stored in the triplestore: there are only so many data sources that can be integrated into it. This limits the scope of analyses possible with the data without additional processing layers. Our SADI-based approach is free from this limitation – thousands of information sources may be wrapped as SADI services and immediately available for ad hoc analyses.

### Query rewriting

An alternative to materialisation is *query rewriting*, when queries in terms of the target semantic schema are converted to equivalent queries in terms of the source relational schema. This approach is very popular and is implemented at least in [9,11,37,40,41,45]. It allows querying live data and requires lighter infrastructure that materialisation: there is no need to set up and maintain a triplestore and data translation utilities. However, it suffers from the same drawback as materialisation, since, by itself, it does not support data integration.

### Relation to data integration

We would like to mention that some of the prior experimental efforts on semantic querying of RDB specifically targeted the data integration task rather than self-service ad hoc querying of isolated RDB. For example, SIMS [36] and TAMBIS [8] – early and well-cited semantic querying projects – were focused on data integration. The integration aspect of semantic querying is especially important in the Clinical Intelligence context because many analyses of clinical data require drawing data from external sources of information about diseases, drugs, infectious agents, medical equipment, etc, as exemplified by the last three of our four use cases.

Currently, publishing relational data as Linked Data seems to be the most popular approach to semantic integration of relational data (see, e.g., [40,41]). For example, the Virtuoso SPARQL engine can crawl the Web by resolving the URIs of RDF resources and using the obtained data together with the underlying relational data to answer SPARQL queries. However, although its Web-like nature makes Linked Data easy to surf, efficient implementation of querying of Linked Data is still a major technical challenge.

Another possibility is federated querying of multiple distributed SPARQL endpoints each representing a separate RDB or data source of a different type. This possibility is currently almost hypothetical because efficient federated querying is difficult: the optimisation methods (mostly inherited from relational databases) that work well on triplestores, do not transfer well to the case of distributed endpoints.

Since SADI is originally designed with the data federation task in mind, it’s not surprising that our SADI-based approach copes well with the integration challenge. Our experiments with Use cases 3-4 suggest that if the external data sources are available via SADI services, the integration is automatic and transparent to the user.

### Temporal reasoning

Although we discuss temporal reasoning in this article primarily to illustrate the capabilities of SADI, in particular the extensibility of our approach, because of the practical importance of the temporal reasoning task, we would like to briefly discuss existing work in this direction. All publications related to SPARQL querying with temporal constraints we have found address the problem of defining mechanisms for specifying validity time of assertions in RDF, and study algorithms that can answer queries extended accordingly. For example, [46], which is also a good source of references on the topic, proposes extensions to RDF and SPARQL, that allow to specify the *validity time* of RDF facts and formulate queries that may require testing or retrieval of temporal coordinates of facts. The article also defines a decision procedure for temporal entailments by reducing them to their non-temporal counterparts, and an algorithm for answering extended SPARQL queries. Similar earlier efforts were presented in [47-49].

Our approach is much more lightweight because we do not aim at processing validity time of arbitrary statements: our SADI services can process any time region descriptions modulo a simple temporal ontology and there are no constraints on the origin of these temporal values or on the way they are attached to the entities they characterise (situations, processes, events). Consequently, our approach does not require any changes to RDF or SPARQL, and requires only minor extensions to the SPARQL implementation. On the other hand, our approach is specific to SADI and may not transfer to other SPARQL implementations.

Temporal arithmetics or more complex temporal reasoning are useful primarily in the context of relatively expressive querying. However, many scenarios related to clinical data analysis do not require much flexibility for making data selections but can benefit from more *interactivity* and *visual analysis* facilities. One paradigm that caters for such needs is *visualisation of temporal categorical data* (see, e.g., [50]). The idea is to identify a limited number of key events associated with a patient, such as procedures of certain types and qualitative findings of medical tests of certain types, and put the events on a *timeline*. Such timelines can be depicted and visually manipulated by users to obtain insights into the patient data based on relative temporal positions of the events and their frequency. This approach is implemented in the Lifelines2 system [50]. Note that visualisation is quite orthogonal to data selection and the possibility of combining semantic querying with timelines-based visualisation, so that the former is used as a flexible means of extracting input data for the latter, poses an interesting research question.

## Conclusions and future work

The main conclusion from our work on semantic querying for Hospital-Acquired Infections surveillance and research so far is that the use of SADI services via a SPARQL interface is a viable general direction in search for a comprehensive solution, although a lot of experimental work remains to be done on the project. In particular, the flexibility of the SADI-based approach stemming from the possibility of using arbitrarily complex programming inside services, makes it sufficiently powerful to cope with problems that seem challenging for approaches using declarative data-to-ontology mappings. We have used temporal reasoning as an example of a problem where the SADI approach excels.

In our future work, when our SADI-based infrastructure is sufficiently mature for comparative studies, we are planning to also try several declarative approaches as a means to semantically query the datawarehouse, such as D2R [40] and *query rewriting*[9,11].

The downside of the SADI approach’s reliance on programming is the relatively high cost compared to declarative approaches: reasonably well designed declarative mappings are easier to create, examine and modify than Java or Perl code of SADI services. In our future work we will look for ways to reduce the cost. A promising direction is to reuse the PSOA RuleML rules, that we currently write only to document the semantic data mapping, for *automatic generation of services*. The core idea is to use Incremental Query Rewriting [11] to automatically “compile” semantic descriptions of SADI services to SQL queries to the HAI datawarehouse, using the semantic mapping rules. This seems feasible at least for simple services. For example, consider the description of *getDiagnosisByPatient*. We assert input class membership for a skolem constant (URI), say *:my_input* :

The output class gives rise to the following query(expressed in PSOA RuleML syntax):

We load the input class assertion and the query together with the rule base encoding the DW semantic mapping and all the referenced ontologies, into a query rewriting reasoner, e.g., the prototype from [11], which should be able to trivially derive the following *schematic answer* for the query, expressed as a PSOA RuleML rule:

The two conditions in lines 5–11 can be represented as the following (parameterised) SQL query:

Assuming that *patWID_by_Patient* denotes the inverse function for *Patient_by_patWID*, the generated service code will replace the parameter “?” with *patWID_by_Patient(input)*, where *input* is the actual input patient URI, before executing the query on the DW. This is justified by lines 12–13 in the schematic answer. Lines 14-15 tell the service code generator what the service should do with the results of the SQL query: the values of *diagnosisID* needs to be converted to URIs with the help of *Diagnosis_by_hdgWID* before they are attached to the input URI via the property *haio:patient_has_diagnosis*.

We are going to develop these ideas in a forthcoming project. In the longer term, we will also evaluate the transferability of our results to other HAI-related settings, e.g., other HAI-related databases, and other applications of Clinical Intelligence, such as adverse drug event monitoring and clinical trial cohort selection.

## Competing interests

The authors declare that they have no competing interests.

## Authors’ contributions

AR developed the semantic mapping from the DW to HAIO and several SADI services, wrote the SPARQL queries and conducted the experiments with Hydra, and wrote the first draft of the article. AK implemented most of the SADI services and conducted the experiments with SHARE. ASN wrote the HAI Ontology. GWR and AJF provided subject matter expertise critical to the development of the ontology and the understanding of the data, and helped to develop the queries. AJF also secured the access to the datawarehouse. DLB identified the use cases and provided insights on the application area. CJOB concieved the idea of using SADI for this application. DLB and CJOB coordinated the project. All authors read and approved the final manuscript.
